# Optimal Line and Tube Placement in Very Preterm Neonates: An Audit of Practice

**DOI:** 10.3390/children4110099

**Published:** 2017-11-17

**Authors:** Daragh Finn, Hannah Kinoshita, Vicki Livingstone, Eugene M. Dempsey

**Affiliations:** 1 Department of Paediatrics and Child Health, University College Cork, Wilton, Cork, Ireland; hmkinos@gmail.com (H.K.); g.dempsey@ucc.ie (E.M.D.); 2 Irish Centre for Fetal and Neonatal Translational Research (INFANT), University College Cork, Cork, Ireland; v.livingstone@ucc.ie

**Keywords:** neonatal, preterm infant, newborn intubation, umbilical venous catheter, umbilical arterial catheter

## Abstract

Background: Placement of endotracheal tubes (ETTs) and umbilical catheters (UCs) is essential in very preterm infant care. The aim of this study was to assess the effect of an educational initiative to optimize correct placement of ETTs and UCs in very preterm infants. Methods: A pre–post study design, evaluating optimal radiological position of ETTs and UCs in the first 72 h of life in infants <32 weeks gestational age (GA) was performed. Baseline data was obtained from a preceding 34-month period. The study intervention consisted of information from the pre-intervention audit, surface anatomy images of the newborn for optimal UC positioning, and weight-based calculations to estimate insertion depths for endotracheal intubation. A prospective evaluation of radiological placement of ETTs and UCs was then conducted over a 12-month period. Results: During the study period, 211 infants had at least one of the three procedures performed. One hundred and fifty-seven infants were included in the pre-education group, and 54 in the post-education group. All three procedures were performed in 50.3% (79/157) in the pre-education group, and 55.6% (30/54) in the post-education group. There was no significant difference in accurate placement following the introduction of the educational sessions; depth of ETTs (50% vs. 47%), umbilical arterial catheter (UAC) (40% vs. 43%,), and umbilical venous catheter (UVC)(14% vs. 23%). Conclusion: Despite education of staff on methods for appropriate ETT, UVC and UAC insertion length, the rate of accurate initial insertion depth remained suboptimal. Newer methods of determining optimal position need to be evaluated.

## 1. Introduction

Endotracheal tubes (ETTs) and arterial and venous umbilical catheters (UCs) are commonly used interventions in the management of preterm and critically ill neonates. Accurate positioning of indwelling ETTs and UCs is essential in providing adequate ventilation, haemodynamic monitoring, fluid and medication administration, and accurate positioning is essential in the avoidance of potentially life-threatening complications.

Despite their necessity in neonatal care, the placement of ETTs and UCs can be difficult, especially for junior medical staff, and success rates only improve as experience is gained [1]. Successful insertion rates for ETTs and UCs are as low as 50% for doctors during the first years of their neonatal training [2,3]. Rates for successful insertion and correct positioning would be lower still. This is important as malpositioned ETTs are associated with hypoxaemia, pneumothorax and right upper lobe collapse [4]. UC malpositioning can cause hepatic necrosis, cardiac arrhythmias, cardiac tamponade and thrombosis [5,6,7]. Adjustment of incorrectly placed ETTs or catheters requires additional handling of the infant, exposure to radiation and potentially increased risk of infection [8,9].

The optimal position of ETT is between the first and third thoracic vertebrae on a chest X-ray. The most widely used equation for estimating insertion depth is calculated by adding 6 cm to the infant’s weight in kg, also known as the‘7-8-9 rule’ [10]. The optimal position of the umbilical venous catheter (UVC) is at the junction of the inferior vena cava (IVC) and right atrium (RA). The high position is recommended for umbilical arterial catheters (UACs); in the aorta, it is ideally between T6 and T9 vertebral bodies [11]. Despite a number of calculations based on both birth weight and external measurements for estimating correct positioning, there is a lack of consensus in the literature as to which is the most accurate [2,9,12,13,14].

The primary aim of this study was to assess the effectiveness of an educational initiative to optimize correct placement of ETT, UVC and UACs in preterm infants less than 32 weeks in a tertiary neonatal unit.

## 2. Methods

Cork University Maternity Hospital (CUMH) is a tertiary teaching and referral hospital with 8500 deliveries annually. Approximately 1400 (16%) of these infants are admitted to the Neonatal Unit (NICU), including 120 infants <32 weeks’ gestation and 100 very low birth weight infants (birth weight <1500 g). The Neonatal Department is a recognized training centre by the Royal College of Physicians, Ireland at Basic Specialist Training (BST) and Specialist Registrar Training (SPR) levels. The Neonatal Medical staff consists of 5 full-time consultant Neonatologists, 9 doctors in BST training and 9 SPR trainees. Currently, there are no neonatal nurse practitioners in the service. The majority of the procedures, such as ETT and UC insertions, are performed either independently for experienced practitioners, or under direct supervision for novice practitioners. All trainees are required to attend the neonatal resuscitation program (NRP). The NRP is a standardized training programme and information relating to ET and UC insertion is included as one part of its syllabus [15]. However, practical procedures are only one component of a comprehensive resuscitation course.

The study design was a pre–post-educational intervention concept. All infants <32 weeks GA admitted to the neonatal intensive care unit (NICU) in CUMH were eligible for inclusion in both the pre- and post-intervention periods. The records of a retrospective cohort of infants <32 weeks’ gestation admitted between January 2010 and October 2012, who required intubation and/or UC insertion, were identified as the pre-intervention group. Following an educational intervention, a prospective cohort of infants <32 weeks’ gestation born between November 2013 and November 2014, requiring one or both of these interventions, was gathered.

Infants were identified from the NICU admission logbooks. All radiographic images taken during the first 72 h of life (abdominal and chest X-rays) were reviewed on the radiology database (IMPAX 6.5.3.562) by two consultant paediatric radiologists. Radiological ETT positions were defined as high (above T1), correct (between T1–T3) or low (below T3). Umbilical venous catheter positions were defined as low (within or below liver), correct (between RA–IVC junction and IVC-diaphragm junction), or high (above RA–IVC). Umbilical arterial catheter positions were defined as high (above T6), correct (between T6–T9) or low (below T9).

The educational intervention was administered to all neonatal doctors during October 2013. Two separate educational sessions were held, and all neonatal BST and SPR trainees attended at least one session. The content of the educational presentations included a presentation of the findings from the retrospective review, which were highlighted to the trainees. The educational content also included information about the importance of correct positioning and the complications associated with malpositioning of ETTs and UCs. In addition, participants were shown surface anatomy images of the newborn, weight-based calculations to estimate insertion depths for endotracheal intubation, and measurements to optimize UC placement. Formal departmental guidelines were also presented and reviewed. ETT insertion depth follows NRP guidelines and is weight based [15]. Estimates for UC insertion depths are based on measurements of surface anatomy. Guidelines for UAC placement are insertion to 1 cm greater than the infants’ umbilical-to-shoulder length. For UVC insertion, the distance between the infants’ umbilicus to the xiphoid process is advised. These guidelines on ETT and UC insertion included detailed instructions, and examples, for ETT, and step-by-step instructions for the appropriate body surface lengths for UC placement. As the initial dataset was collected retrospectively, we were unable to determine which procedures were elective and which were performed as an emergency.

The aim of the sessions was not only to educate all the junior and senior staff, but also to try and standardise the approach, which despite guidelines, may have been variable in the past. These guidelines were made available in all clinical areas. All trainees were still required to have attended a NRP and the training was designed as a complement to the NRP syllabus. All trainees continued to be supervised by a consultant for all procedures until deemed independently competent. The Cork University Hospitals Research Ethics Committee approved this study (Ethical approval code: ECM4u031213). Ethical approval was Granted by both Cork University Maternity Hospital and University College Cork Research Ethics Committees’.

### Statistical Analysis

The two cohorts were statistically described and categorized with respect to birth weight and GA. For comparisons between the pre- and post-education groups, the independent samples t-test was used for continuous variables and the χ^2^ test or Fisher’s exact test (in the case of small expected counts) was used for categorical variables. Multiple logistic regression analysis was performed to investigate differences between the two groups in terms of correct placement after controlling for the potential confounding effects of gestational age and birth weight. All tests were two-sided and a *p*-value < 0.05 was considered to be statistically significant. IBM SPSS Statistics (Version 22) was used for the statistical analysis.

## 3. Results

During the two study periods, 211 infants <32 weeks GA had an ETT and/or UC inserted during the first 72 h of life, and were therefore eligible for inclusion in this study. One hundred and fifty-seven infants were included in the pre-education group, and 54 in the post-education group. All three procedures were performed in 50.3% (79/157) in the pre-education group, and 55.6% (30/54) in the post-education group.

GA differed between pre- and post-study groups for the UAC intervention (*p* = 0.029) and the UVC intervention (*p* = 0.036), with the average GA slightly higher for infants in the post group (Table 1). A statistically significant difference between pre and post groups was also found for birth weight for the UVC intervention (*p* = 0.022), with the average birth weight higher for infants in the post group (Table 1).

**Figure 1:**
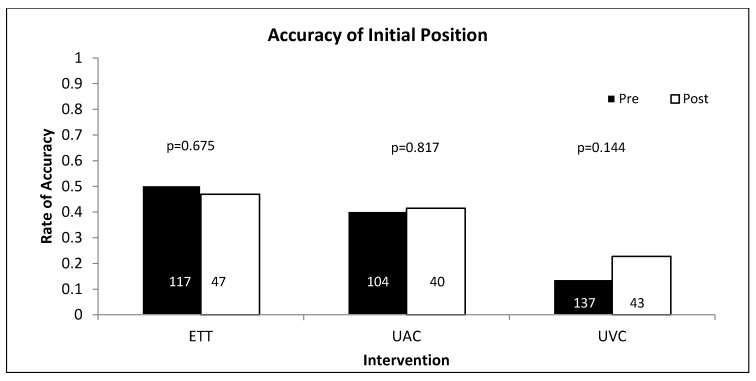
Accuracy of initial position in pre (black) and post (white)-education groups.

There were no statistically significant differences in positioning success between the pre- and post-education groups (Figure 1). ETT position was correct on initial positioning in 50% (59/117) of cases in the pre-education group and 47% (22/47) in the post-education group (*p* = 0.675). UACs were correctly positioned in 40% (42/104) in the pre-education group and 43% (17/40) in the post-education group (*p* = 0.817). UVCs were correctly positioned in 14% (19/137) in the pre-education group and 23% (10/43) in the post-education group (*p* = 0.144). Adjustment for gestational age and birthweight did not alter the conclusions.

**Figure 2:**
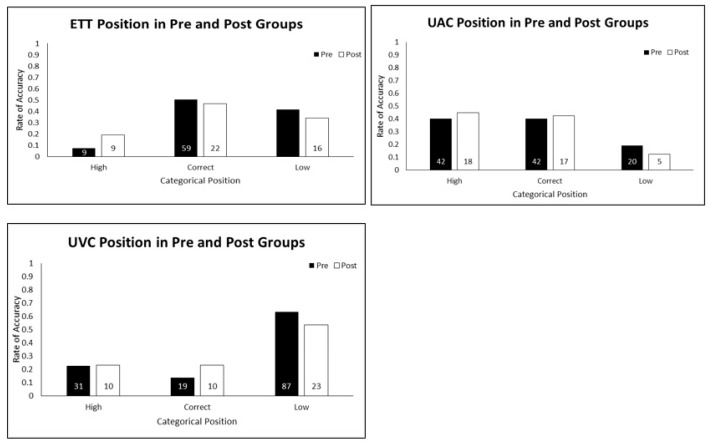
Accuracy of initial position for ETT, UAC and UVC in pre and post groups.

The ETT and UC positions were further categorized as high, correct or low (Figure 2). There were no statistically significant differences in positioning between the pre- and post-education groups (ETT *p* = 0.099, UAC *p* = 0.627 and UVC *p* = 0.311) for this categorization. A relative decrease of low positioning of ETT (42% vs. 34%, *p* = 0.356), UAC (19% vs. 13%, *p* = 0.340) and UVC (64% vs. 54%, *p* = 0.240) from pre- to post-education groups was found but the differences were not statistically significant.

Initial positioning success for each intervention was also assessed according to GA and birth weight. For ETT, no statistically significant differences were found for either GA (*p* = 0.809) or birth weight (*p* = 0.335) (Table 2). For the UAC intervention, statistically significant differences were found for both GA (*p* = 0.006) and birth weight (*p* = 0.005), with mean GA and birth weight higher in the correctly positioned group. Gestational age was also significantly higher in the correctly positioned group for the UVC intervention (*p* = 0.013) and there was a tendency towards infants in the correctly positioned group to have higher birth weights, but this failed to reach statistical significance (*p* = 0.054).

## 4. Discussion

In this study of 211 infants <32 weeks GA, successful positioning of ETTs and UCs did not change after an educational intervention. Successful positioning was highest with ETT placements, with just half of all radiographs showing a satisfactory initial position. However, UACs were inserted to a safe recommended position in only 40% of cases while the UVC was positioned correctly in less than a quarter of infants, both before and after the intervention. The reason for low successful baseline rates in all three procedures may represent the inaccuracy of the current guidelines and the failure of the educational intervention may reflect the failure of these estimated measurements, the content or delivery of the educational intervention or the poor retention by the participants. It is difficult to determine which is the underlying cause, or if all are contributory. Our findings should be generalizable to neonatal units of similar size and structure, but limited by the calculations we used for depth of insertion.

Malpositioning rates of 40–50% for ETT placements in VLBW infants have been reported, which are consistent with our findings [16,17,18,19]. The most widely used equation for estimating the appropriate depth of ETT insertion is the 7-8-9 rule based on the infant’s weight [16]. However, this rule may overestimate insertion depth for extremely preterm infants, especially for ELBW infants. In a study by Jain et al., pre-study successful positioning was reported as 48% with a weight-based normogram-estimating depth of insertion. However, in their prospective cohort, correct positioning occurred in >85% of infants for both the control and study (suprasternal palpation) groups [20]. Therefore, correct positioning rates for oral ETT placement in VLBW infants can be achieved with existing formula. This implies that for ETT placements, weight-based normograms may be appropriate for initial positioning. This would suggest that, in this instance, the educational intervention for ETT positioning may have failed. It is important to note that current international guidelines recommend confirmation of ETT position with CO_2_ detectors. However, malpositioning will only be ascertained by a chest X-ray, and should be considered prior to surfactant delivery if not deemed clinically urgent. Previous reports of UC positioning have also had low success rates. Dunn published graphs based on umbilicus to shoulder measurements after assessing 50 infants on postmortem [12]. Shukla and Wright have produced separate mathematical estimates of depth insertion based on birthweight [9,13]. O’Donnell et al. performed a RCT comparing estimates based on surface anatomy and birthweight [3]. Methods were equivocal for UVC insertion and resulted in 30% success rates, which are in keeping with our study finding. UAC positioning, when surface anatomy measurements were used, was similar to our cohort and successful 50% of the time. However, for UAC positioning, formulas based on birthweight had much higher success rates in the region of 90%. A RCT performed by Gupta et al. found success rates of 57% for both UVC and UAC based on surface anatomy Shukla graphs [21]. UVC and UAC correct positioning rates of 94% and 92% respectively were achieved when surface anatomy measurements based on umbilical to nipple/pubis symphysis were used. These success rates are promising but as four different surface anatomy measurement groups were incorporated into the study, there were small study numbers in each group and further studies are warranted. Overall, a formula to accurately calculate insertion depth for UCs in extremely preterm infants has remained elusive and success rates remain low. These findings suggest that new guidelines for estimating insertion depths are required.

The educational approach in our study was aimed at addressing optimal positioning of the ETT or UC. We did not concentrate on tube insertion techniques nor did we address the number of attempts at insertion. Instead, our focus was on standardizing the estimation techniques for depth of insertion for each procedure. Educational sessions were held and ensured that all members of the neonatology team were using the same measurement techniques. The educational sessions also heightened awareness of the importance of correct measurements. Performing a pre-study audit also allowed us to understand our baseline success rates, and highlight these low rates during educational sessions.

The educational intervention was unsuccessful in that no improvement was identified. However, we cannot infer from our results whether the calculations used, the format of the intervention, or poor retention of knowledge and skills was responsible. Studies on practical procedures have shown that mannequin simulation training leads to superior in-hospital results when compared to traditional methods [22]. Significant improvement in real-life intubation skills has been observed in the period immediately following simulation training as measured by successful intubations [23]. However, some concerns have been raised that simulation-based improvement is short-lived and that real patient experience has a more lasting effect on performance [23]. Retention of knowledge and skills following simulated resuscitation training is also uncertain [24], and educational simulations may not have improved our outcomes. Smartphone apps available at the time of procedure, which can perform calculations and display demonstrations may have a role in the future but further validation is required for such applications [25]. Educational sessions also need to focus on the expectation that the success rates of initial positioning are low, and how to safely manage expectations, such as considering an X-ray pre-surfactant or PICC insertion instead of UVC.

Anatomical variations may mean that a formula, whether based on birthweight or surface anatomical measurements, may not be the most appropriate method for positioning ETTs or UCs in preterm infants. A number of studies have found ultrasound to be superior in identifying the position of UC tip compared with chest X-ray [26,27,28], and real-time ultrasound during positioning is feasible and would be an ideal tool [29]. Ultrasound can also visualize the anatomic position of the ETT position in term and preterm infants [30]. Ultrasound is available in most tertiary neonatal units. However, larger studies and further validation is required prior to the introduction of US for placement of ETTs and UCs in preterm infants. Also, although many experienced neonatologists will be familiar with US as a result of performing cranial ultrasounds, US for this purpose will require further training for neonatal staff.

There were a number of limitations to this study. Firstly, a power calculation was not performed and therefore we may have missed positive findings that would have been present if we had recruited a larger prospective cohort. This is unlikely due to the nature of our intervention and loss of knowledge over time. If there were a positive finding, we believe it should have been evident within our study timeframe. Secondly, neonates in the post group were slightly more mature and had a larger mean birth weight. We did not obtain data about the experience of the individuals performing the procedure and therefore are not able to compare outcomes across different levels of training and experience. It would have been useful to objectively measure the effectiveness of the educational period and retention of information in the doctors performing these procedures. Without this data, it is not possible to determine whether the low rates of accuracy can be attributed to the methods and formulae to calculate a recommended depth of insertion, physician training/experience, or other extraneous factors such as the level of emergency of the situation. Lastly, the intervention was designed around traditional teaching techniques, and did not take advantage of newer teaching methods, which could have incorporated simulation training or the use of smartphone applications.

In conclusion, this study did not identify improvements in ETT, UVC and UAC positioning following a traditional educational initiative. Our findings have highlighted a significant need for ongoing quality improvement in the training and execution of ETT and UC placement. Educational simulation sessions may enhance traditional teaching methods, and smartphone applications may have a role in the future. Furthermore, our findings highlight the need for revised formulae to calculate insertion depth of ETTs and UCs in preterm neonates.

## Figures and Tables

**Table 1:** Infant gestational age and birth weight in pre and post education groups.

		GA		Birth Weight (g)	
Group (N)		Mean (SD)	*p*-Value	Mean (SD)	*p*-Value
ETT	Pre Group (117)	27.09 (2.05)	0.062	1012 (323)	0.342
	Post Group (47)	27.77 (2.20)		1071 (422)	
UAC	Pre Group (104)	26.67 (1.82)	0.029	922 (237)	0.153
	Post Group (40) ^1^	27.48 (2.28)		996 (362)	
UVC	Pre Group (137)	27.28 (2.01)	0.036	985 (274)	0.022
	Post Group (43) ^2^	28.05 (2.24)		1106 (366)	

^1^ *n* = 39 for birth weight; ^2^ *n* = 42 for birth weight. Abbreviations: ETT—endotracheal tube; UAC—umbilical arterial catheter; UVC—umbilical venous catheter.

**Table 2:** Comparison of GA and birth weight between incorrect and correct positioning.

		GA		Birth Weight (g)	
Group (N)		Mean (SD)	*p*-Value	Mean (SD)	*p*-Value
ETT	Incorrect (83)	27.24 (2.06)	0.809	1002 (361)	0.335
	Correct (81)	27.32 (2.17)		1056 (347)	
UAC	Incorrect (85)	26.52 (1.84)	0.006	889 (269)	0.005
	Correct (59) ^1^	27.44 (2.06)		1020 (273)	
UVC	Incorrect (151)	27.30 (2.07)	0.013	994 (287)	0.054
	Correct (29) ^2^	28.34 (1.99)		1112 (357)	

^1^ *n* = 58 for birth weight; ^2^ *n* = 150 for birth weight.

## Abbreviations

BST	basic specialist trainee
ETT	endotracheal tube
GA	gestational age
IVC	inferior vena cava
NICU	neonatal intensive care unit
RA	right atrium
SPR	specialist registrar trainee
UAC	umbilical arterial catheter
C	umbilical catheter
UVC	umbilical venous catheter
